# Biomarker Measurements in a Coastal Fish-Eating Population Environmentally Exposed to Organochlorines

**DOI:** 10.1289/ehp.7970

**Published:** 2005-06-01

**Authors:** Pierre Ayotte, Éric Dewailly, George H. Lambert, Sherry L. Perkins, Raymond Poon, Mark Feeley, Christian Larochelle, Daria Pereg

**Affiliations:** 1 Unité de Recherche en Santé Publique, Centre Hospitalier Universitaire de Québec, Université Laval, Québec, Canada; 2 Laboratoire des Biomarqueurs, Institut National de Santé Publique du Québec, Québec, Canada; 3 Environmental and Occupational Health Sciences Institute, Robert Wood Johnson Medical School, University of Medicine and Dentistry of New Jersey, Piscataway, New Jersey, USA; 4 Division of Biochemistry, Ottawa Hospital, Ottawa, Ontario, Canada; 5 Environmental Health Science Bureau, and; 6 Bureau of Chemical Safety, Health Canada, Ottawa, Ontario, Canada

**Keywords:** cytochrome P450 CYP1A2, d-glucaric acid, dioxins, enzyme induction, food chain, organochlorine insecticides, polychlorinated biphenyls, porphyrins, smoking

## Abstract

The Lower North Shore region of the St. Lawrence River is home to a fish-eating population that displays an unusually high body burden of several organochlorines, including polychlorinated biphenyls (PCBs) and dioxin-like compounds (DLCs). We measured biomarkers indicative of liver enzyme induction and investigated the relationship with organochlorine body burden in adult volunteers from this population. We determined plasma concentrations of PCBs and chlorinated pesticides by high-resolution gas chromatography (HRGC) with electron capture detection. DLC concentrations were measured by the dioxin-receptor chemically activated luciferase expression (DR-CALUX) assay and in a subset of participants, by HRGC/high-resolution mass spectrometry. We measured cotinine, d-glucaric acid, and porphyrins in morning urine samples and determined liver CYP1A2 activity *in vivo* using the caffeine breath test. Neither DLC concentrations as measured by the DR-CALUX nor PCB-153 concentrations, the latter representing total PCB exposure, were correlated with biomarkers of effects. Smoking (morning urinary cotinine concentration) was positively related to CYP1A2 activity as measured by the caffeine breath test (*p* < 0.01). Liver CYP1A2 activity was in turn negatively correlated with PCB-105:PCB-153 and PCB-118:PCB-153 congener ratios (*p* < 0.05). Hence, despite the relatively high body burden of PCBs and DLCs in this population, only smoking had a significant correlation with biomarkers of hepatic enzyme induction. Our data are consistent with smoking-induced liver CYP1A2 activity altering heme metabolism and increasing the biotransformation of mono-*ortho* PCB congeners.

Organochlorines (OCs) constitute a family of persistent, lipid-soluble compounds that includes industrial chemicals [e.g., polychlorinated biphenyls (PCBs), hexachlorobenzene (HCB)], pesticides (e.g., DDT, methoxychlor, mirex), and by-products of combustion and various industrial processes [e.g., polychlorodibenzo-*p*-dioxins (PCDDs) and polychlorodibenzofurans (PCDFs)]. These compounds are released into the environment at southern latitudes and transported to northern regions by long-range oceanic and atmospheric transport processes (Barrie et al. 1992; Macdonald et al. 2000). OCs bio-accumulate in adipose tissues of various species from northern aquatic ecosystems. Biomagnification occurs in food webs, leading to relatively elevated concentrations in species located at the highest trophic levels, including human populations that consume large amounts of sea products (Dewailly et al. 1993; Kiviranta et al. 2002; Sjödin et al. 2000).

The Lower North Shore of the St. Lawrence River, a remote coastal region of Québec, consists of 15 communities spread over a 400-km shoreline extending from Kegaska to Blanc Sablon (Figure 1). A large proportion of the 6,000 residents relies on fishing for subsistence and consequently consumes large amounts of seafood (Dewailly et al. 1992). Results from surveys conducted since 1990 have indicated that this population is highly exposed to PCBs and to dioxin-like compounds (DLCs) such as 2,3,7,8-substituted PCDDs and PCDFs as well as nonsubstituted and mono-*ortho*–substituted PCBs, compared to the southern Québec population (Dewailly et al. 1992; Muckle et al. 1998; Ryan et al. 1997). Food items that contribute the most to this exposure are sea-bird eggs and possibly cod and seal liver (Dewailly et al. 1992; Ryan et al. 1997).

Exposure to DLCs produces a wide variety of biologic and toxic effects such as teratogenesis, immunosuppression, and tumor promotion, most of them dependent on the activation of the aryl hydrocarbon receptor (AhR) (Mimura and Fujii-Kuriyama 2003; Poland and Knutson 1982; Safe 1990; Van den Berg et al. 1998). We previously reported on a group of 25 individuals from the Lower North Shore of the St. Lawrence River that had a mean total DLC concentration of 250 ng of 2,3,7,8-tetrachlorodibenzo-*p*-dioxin (TCDD) toxic equivalents (TEQ)/kg plasma lipids, including PCDDs, PCDFs, and non-substituted and mono-*ortho* PCBs (Ryan et al. 1997). Assuming a 20% body fat content in humans, the corresponding body burden would be 50 ng TEQ/kg body weight. In laboratory animals exposed to TCDD, adverse effects (hormonal, reproductive, and developmental) have been observed at body burdens in the range of 28–73 ng TCDD/kg body weight (van Leeuwen et al. 2000), hence suggesting that DLC exposure in this fish-eating population might induce adverse health effects.

To refine the assessment of the health risks possibly related to the body burden of DLCs and other OCs in this fish-eating population, investigators can measure early biologic events such as the induction of drug-metabolizing enzyme activities. Activation of genes coding for biotransformation enzymes such as cytochrome P4501A1 (CYP1A1) and CYP1A2 is a well-known consequence of TCDD binding to the AhR (Walker et al. 1999; Whitlock 1999). Liver CYP1A2 activity in humans can be measured *in vivo* by a noninvasive method, the caffeine breath test (CBT; Kotake et al. 1982). In this test, CYP1A2 activity is monitored by following the appearance of ^13^CO_2_ in exhaled breath resulting from the oxidative demethylation of [3-^13^C-methyl]caffeine (Abraham et al. 2002; Brambilla et al. 1995; Kotake et al. 1982; Lambert et al. 1990). The concentration of d-glucaric acid in urine has also been proposed as a biomarker of exposure to xenobiotics (Brewster 1988; Hogue and Brewster 1991; Poon et al. 1993). Glucuronidation is a major biotransformation pathway for a wide variety of xenobiotics and drugs, and its induction results in increased excretion of d-glucaric acid (Brewster 1988). Urinary d-glucaric acid excretion has also been shown to increase in humans exposed to OC pesticides (Hunter et al. 1972; Notten and Henderson 1977), PCBs (Maroni et al. 1984), and TCDD (Ideo et al. 1982). Urinary porphyrin excretion can also be altered by exposure to xenobiotics such has PCBs (Colombi et al. 1982), polybrominated biphenyls (Strik et al. 1979), and TCDD (Hoffman et al. 1986; Jung et al. 1994). Provided sufficient exposure dose, all these chemicals induce chronic disturbances in hepatic synthesis of porphyrins and thus lead to excess total porphyrin excretion and skin symptoms in the final stage (Hogue and Brewster 1991). Presumably, these chemicals would also alter urinary porphyrin profile at early stages before overt toxicity, and therefore, the latter constitutes a much more sensitive biomarker than total porphyrin determination (Johnson et al. 1988).

In the present study, we measured concentrations of porphyrins and d-glucaric acid in urine samples and performed the CBT in volunteers from two communities located on the Lower North Shore of the St. Lawrence River. We measured plasma lipid concentrations of PCBs and chlorinated pesticides by high-resolution gas chromatography (HRGC) with electron capture detection (ECD) and DLCs by the dioxin-receptor chemical-activated luciferase gene expression (DR-CALUX) bioassay. Finally, because tobacco smoking can induce liver enzyme activities, smoking status was assessed by questionnaire and validated using urinary cotinine measurements.

## Materials and Methods

### Study population and measurements.

**Figure 1:**
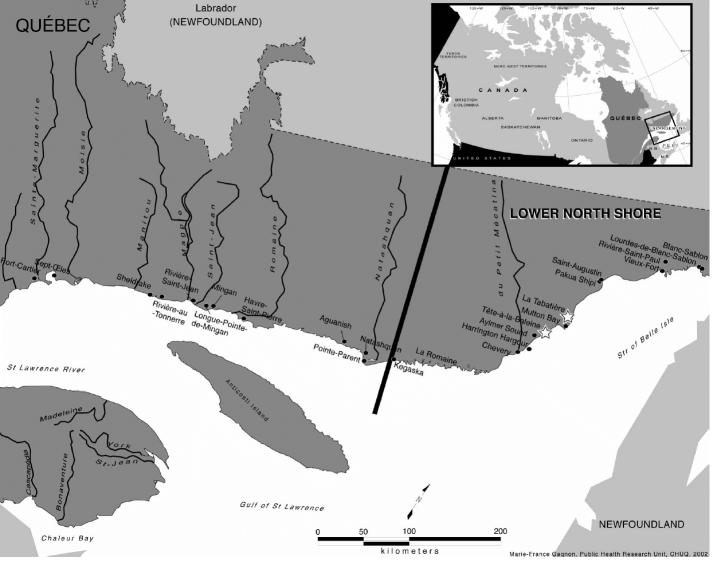
The Lower North Shore region of the St. Lawrence River (Québec, Canada).

We recruited participants in this study from two Lower North Shore settlements: Tête-à-la-Baleine and La Tabatière (Figure 1). Potential subjects were selected from the list of those who participated in a previous survey (Dewailly et al. 1992), and therefore, their concentration of OCs in plasma lipids was already known. We tried to recruit individuals with both high and low exposure to PCBs. Our research nurse contacted potential subjects by telephone and asked interested individuals to visit their local health center the week after for an information meeting. After a brief medical examination, subjects with cardiac arrhythmia, uncontrolled hypertension, or with a history of caffeine intolerance were excluded from the study. An 80-mL venous blood sample was drawn from a cubital vein and collected in Vacutainer (Becton Dickinson, Franklin Lakes, NJ, USA) tubes (10 mL) containing EDTA as the anticoagulant. Tubes were centrifuged (10 min) immediately after blood sampling, and plasma samples were kept frozen (−15°C) in plain Venoject tubes (10 mL) until OC analysis at the Laboratoire de Toxicologie (Institut National de Santé Publique du Québec, Québec, QC, Canada). Volunteers were also asked to collect their first urine on the next morning in a plastic vial containing sodium bicarbonate (0.5 g/10 mL urine). Urine samples were kept frozen (−15°C) until analysis for d-glucaric acid and cotinine (Environmental Health Science Bureau, Health Canada, Ottawa, Ontario, Canada) and porphyrins and creatinine (Division of Biochemistry, Ottawa Hospital, Ottawa, ON, Canada). Finally, volunteers were asked to fast for 6 hr and not to drink or eat methylxanthine-containing foods (chocolate, coffee, tea, cola) for 12 hr before the CBT, which was conducted the next morning.

We obtained anthropometric measurements (weight, height), and administered a short questionnaire to obtain information on current medication and smoking and drinking habits. We obtained informed consent from participants before administering the questionnaire and collecting biologic samples. This study was approved by the Ethics Committee from Laval University Medical Center (Centre Hospitalier Universitaire de Québec).

### Caffeine breath test.

The CBT was administered as previously described by Kotake et al. (1982). Briefly, [3-^13^C-methyl]caffeine (Cambridge Isotopes, Cambridge, MA, USA) dissolved in water (10 mg/mL) was administered orally at a dose of 3 mg/kg body weight (up to a maximum of 200 mg). Subjects were asked to remain seated for 15 min before the test and during the 2-hr period after caffeine administration. Before drinking the caffeine solution, volunteers exhaled into an air collection device made of a modified 20-mL syringe. This device allows the subject to exhale the air contained in the lower respiratory tract and to trap the alveolar air that is needed for the CO_2_ analysis. Air samples were also collected at 30, 60, 90, and 120 min postcaffeine administration. Duplicate samples were taken at each time. Air samples (16 mL) were transferred into evacuated glass containers for transport and storage to the laboratory. After cryogenic purification of the CO_2_ present in exhaled air samples, the ^13^CO_2_:^12^CO_2_ ratio was determined by differential gas-isotope ratio mass spectrometry according to Schoeller and Klein (1979). The quantity of labeled CO_2_ exhaled was expressed as percent labeled dose exhaled per hour (Schneider et al. 1978). The excess ^13^CO_2_-exhaled/mmol ^12^CO_2_ for the dose was determined and multiplied by the basal CO_2_ production rate (assuming 300 mmol/m^2^ of body surface per hour) (Schneider et al. 1978; Schoeller and Klein 1979). Results were expressed as the 2-hr cumulative exhalation of labeled CO_2_, because it is the best monitor of caffeine clearance in the adult (Kotake et al. 1982).

### Urine analyses.

Porphyrins (hepta-, hexa-, pentacarboxylic porphyrins; uroporphyrins; and coproporphyrins) in urine samples were measured by high-performance liquid chromatography with a fluorescence spectrophotometer (Johnson et al. 1988). The detection limit is 0.5 pmol (25 nmol/L). The within-run coefficients of variation for 25 nmol/L and 300 nmol/L were 4–12% and 3–5%, respectively.

d-Glucaric acid was measured by high-performance liquid chromatography with an ultraviolet detector after pretreatment of urine with boronic acid gel to remove interfering substances such as l-ascorbic acid and d-glucuronic acid (Poon et al. 1993). The detection limit of this method is 10 μmol/L d-glucaric acid, and the run-to-run precisions were 9.1 and 7.7% at concentrations of 60 and 310 μmol/L, respectively.

Smoking has been shown to induce CYP1A2 activity in humans. Kotake et al. (1982) reported that the 2-hr cumulative exhalation of ^13^CO_2_ for smokers was almost 2-fold the value for nonsmoking controls. We measured cotinine concentration in urine samples by an ELISA method (COTI-TRAQ, Serex Inc., NJ, USA). Creatinine concentration was determined by a modified Jaffe reaction, using an automated colorimetric analyzer.

### Plasma OC analyses.

Concentrations of ten chlorinated pesticides or metabolites (aldrin, dieldrin, *cis*-chlordane, *trans*-chlordane, *p,p*′-DDE, HCB, mirex, *cis*-nonachlor, *trans*-nonachlor, oxychlordane) and 13 PCB congeners (IUPAC nos. 28, 52, 99, 101, 118, 128, 138, 153, 156, 170, 180, 183, 187) were determined by HRGC/ECD. Plasma samples were mixed with an aqueous solution of ammonium sulfate and ethanol (1:1) and then extracted with hexane. Extracts were concentrated and cleaned by elution through two columns containing activated Florisil. We performed the analysis by dual-column gas chromatography, using an HP 5890 gas chromatograph equipped with two capillary columns (HP Ultra 1 and Ultra 2, both 50 m long, 0.2-mm inner diameter, 0.33-μm film thickness) and twin electron capture detectors (Hewlett Packard, Palo Alto, CA, USA). We achieved identification and quantification of the specific congeners by comparing responses and retention times with calibration standards for each analyte of interest. Recovery of OCs varied between 90 and 103%. The detection limit of the method was 0.05 μg/L for all compounds. The within-run coefficients of variation for PCB congeners and chlorinated pesticides ranged from 5.1 to 13.5% (at 0.4 μg/L).

OC concentrations were expressed on a plasma lipid basis because blood samples were not collected after a fasting period, which may affect plasma OC concentrations (Phillips et al. 1989). To adjust OC concentrations on a lipid basis, we used standard enzymatic procedures to determine total and free cholesterol and triglycerides. We measured phospholipids containing choline by the enzymatic method of Takayama et al. (1977), using a commercial kit (Wako Pure Chemicals Industries, Richmond, VA, USA). We estimated total plasma lipids (TL) by adding the concentrations of cholesterol esters [obtained by subtracting free cholesterol from total cholesterol (TC)], free cholesterol (FC), triglycerides (TG), and phospholipids (PL), according to the following formula (Phillips et al. 1989):


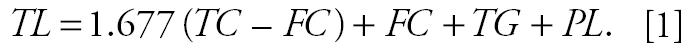


Quantification of 2,3,7,8-chloro-substituted PCDD/PCDF congeners and coplanar non-*ortho* PCB congeners (IUPAC nos. 77, 126, and 169) in plasma samples was performed by AXYS Analytical Services Ltd. (Sydney, BC, Canada). A 5–10 g plasma sample was spiked with labeled surrogate standards (^13^C-labeled analogs of the target PCDDs, PCDFs, and coplanar PCBs) and extracted with an ethanol, hexane, and saturated ammonium sulfate mixture (1:1:1), and the hexane extract was washed with concentrated sulfuric acid and distilled water. The extract was then dried over anhydrous sodium sulfate and cleaned up using the following column chromatography procedures: *a*) layered acidic-basic silica gel column, eluted with hexane; *b*) activated alumina column, eluted with hexane (discarded) followed by elution with 1:1 dichloromethane: hexane (collected); *c*) carbon on celite column (4.75% carbon), eluted with hexane (discarded), followed by 1:1 dichloromethane: cyclohexane and then 10:1 ethyl acetate:toluene (collected together for coplanar PCB congener analysis), followed by inversion of the column and elution with toluene (for collection of PCDDs and PCDFs); *d*) activated alumina column, each fraction (coplanar PCB and PCDD/PCDF) applied to a new alumina column and eluted with hexane (discarded) and 1:1 dichloromethane:hexane (collected for gas chromatograph/mass spectrometry analysis). In preparation for gas chromatograph/mass spectrometry analysis, an aliquot of ^13^C-labeled recovery standard was added to each fraction collected. Each fraction was analyzed by HRGC/high-resolution mass spectrometry (HRMS) using a VG Autospec Ultima high-resolution mass spectrometer equipped with an HP5890 gas chromatograph (Hewlett Packard, Palo Alto, CA, USA). The chromatographic separation was carried out using an Agilent/J&W DB-5 capillary column (60 m long, 0.25 mm inner diameter, 0.1 μm film thickness; Agilent Technologies Canada Inc., Mississauga, ON, Canada).

### DR-CALUX assay.

Plasma samples (2 mL) were mixed with an aqueous solution of ammonium sulfate and ethanol (1:1) and then extracted with hexane. Extracts were concentrated and cleaned by elution through two columns containing activated Florisil. The cleaned extracts were dissolved in dimethyl sulfoxide (DMSO) for CALUX measurements in 96-well plates using H4IIE.Luc cells (kindly donated by A. Brouwer, Vrije Universiteit, Amsterdam, the Netherlands). Cells were grown in Dulbecco’s modified Eagle medium with 10% fetal calf serum (Wisent Inc., St. Bruno, QC, Canada) in 96-well cell culture plates (Sarstedt Inc., Montreal, QC, Canada) to 60–80% confluence and exposed during 24 hr in triplicate to the plasma extracts, TCDD standards (AccuStandard, New Haven, CT, USA), or DMSO alone (vehicle; final concentration, 0.5% vol/vol). Cells were then washed with PBS, lysed in 50 μL lysis buffer (Passive Lysis 5× buffer; Promega, Madison, WI, USA) for 30 min, and the lysate was frozen at 80°C. For measurement of luciferase activity, samples were thawed, and 20 μL lysate was pipetted into a 96-well microtiter plate (Dynex Technologies Ltd., Worthing, UK). The plate was placed in a Lmax luminometer (Molecular Devices Corporation, Sunnyvale, CA, USA), and the following sequence of events was programmed for each well: *a*) injection of 100 μL luciferin assay mix (Promega); *b*) 4-sec delay; *c*) light production measured over a 10-sec period. The limit of detection was approximately 5 pg TEQ/g lipid for a 2-mL plasma sample containing 7 g lipids/L.

### Statistical analysis.

Distributions of values for OC concentrations as well as urinary glucaric acid and porphyrins concentrations were skewed. Hence, we included the median in descriptive statistics. We limited statistical analyses to contaminants that were detected in > 50% of the samples. For contaminants and urinary measurements, a concentration equal to half the detection limit was assumed for samples with concentrations below the detection limit. Relations between biomarkers and contaminants were assessed using Spearman’s correlation coefficients. We used the Mann-Whitney *U*-test to compare biomarker values between age groups (≤45, > 45 years), smoking categories (smokers, nonsmokers), and alcohol consumption categories (occasional drinkers, abstainers). Nonparametric analyses were selected because of the skewed distributions and the small sample sizes. The level of statistical significance was set at 0.05. We used the SPSS for Windows statistical software package (version 11.5; SPSS Inc., Chicago, IL, USA) to perform all statistical analyses.

## Results

### Characteristics of participants.

Forty volunteers (23 women, 17 men) were recruited in the two Lower North Shore communities. The mean age of the participants was 47 years (range, 25–75 years). Ten subjects were on continuous medication at the time of the study, including two taking anticonvulsants (phenobarbital and phenytoin). The group consisted of 17 smokers and 23 nonsmokers, as determined by self-declared cigarette consumption and urinary cotinine results (urine cotinine concentration > 0.8 μg/mL). Fourteen participants drank alcohol occasionally, whereas 26 were abstainers. Mean body mass index was 27.0 (SD = 4.0).

### Biomarkers of exposure.

Plasma concentrations of the various OCs that were detected in most participants are presented in Table 1. Major PCB congeners were congeners 153, 180, and 138. These three congeners represented 68% of the total PCB concentration in plasma lipids. Simple correlation analysis revealed strong associations (Spearman’s *r* > 0.78; *p* < 0.001) between PCB-153 concentrations and those of congeners 99, 138, 156, 170, 180, 183, and 187. Correlations between PCB-153 concentrations and those of mono-*ortho* PCBs 105 and 118 were somewhat lower (Spearman’s *r* = 0.52 and 0.58, respectively; *p* < 0.001). Among chlorinated pesticides, *p*,*p*′-DDE was the most abundant compound in plasma, followed by HCB, *trans*-nonachlor, oxychlordane, and mirex, which showed much lower concentrations (> 10-fold).

**Figure 2:**
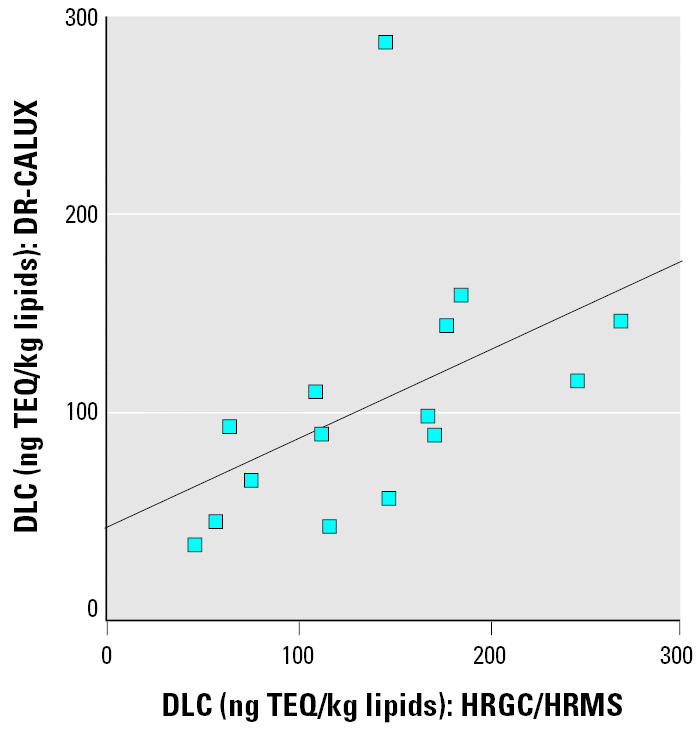
Correlation between concentrations of DLCs in plasma lipids determined by the DR-CALUX bioassay and those determined by HRGC/HRMS. r = 0.66; p = 0.008

DLC concentrations determined by the DR-CALUX assay were moderately correlated with total PCB concentrations (Spearman’s r = 0.45; p = 0.003). Similar correlations were noted between DLC concentrations and either PCB-153 concentrations or total concentrations of mono-ortho congeners (105, 118, and 156) expressed in nanogram TEQs per kilogram plasma lipids. In a subset of participants (n = 15), concentrations of PCDDs/PCDFs and non-ortho coplanar PCBs were measured by HRGC/HRMS and expressed as total TEQ concentrations using the World Health Organization’s toxic equivalency factors (Van den Berg et al. 1998). The latter was significantly correlated with the TEQ concentration measured by the DR-CALUX assay in the same samples (r = 0.66; p = 0.008; Figure 2).

### Biomarkers of hepatic enzyme induction.

Results for hepatic enzyme induction biomarkers are presented in Table 2. d-Glucaric acid was detected in urine samples from 95% of participants (38 of 40). The coefficient of variation (CV) exceeded 100% for this parameter, indicating that the distribution was highly skewed. Two participants, one being treated with phenobarbital and another with phenytoin, exhibited the two highest concentrations (26.0 and 14.9 mmol/mol creatinine, respectively). Eliminating these two values reduced the mean from 4.4 to 3.6 mmol/mol creatinine and the CV to 61%.

Coproporphyrins were present in all urine samples, whereas uroporphyrins were above the limit of detection in only 15% of samples (6 of 40). Penta-, hexa-, and heptaporphyrins were not detected in any urine sample. The distribution of coproporphyrin concentrations was slightly skewed. The highest value was observed for the individual taking phenobarbital. Eliminating the two subjects on anticonvulsant medication slightly reduced the mean (from 11.5 to 11.1 μmol/mol creatinine) and the CV (from 30 to 28%).

The CBT was successfully administered to 21 participants. This parameter followed a normal distribution with the mean very close to the median value and a CV of 41%. Only one subject that underwent the CBT was on antiepileptic medication, and eliminating this subject did not substantially modify the statistical descriptors of the distribution.

We examined relations between biomarkers of effects and found that urinary d-glucaric acid concentrations were weakly correlated with urinary coproporphyrin concentrations (Spearman’s r = 0.36; p = 0.02), whereas the latter were moderately correlated with the CBT (r = 0.57; p = 0.006). d-Glucaric acid concentrations were not correlated with CBT results.

### Biomarkers of exposure versus biomarkers of hepatic enzyme induction.

We explored the relationship between biomarkers of exposure and biomarkers of hepatic enzyme induction. To this end, we used correlation analyses after excluding results from the two individuals taking anticonvulsants, in view of the known enzyme-inducing effects of these agents (phenobarbital and phenytoin). Urinary cotinine concentration was positively related to the CBT (Table 3). We selected PCB-153 to represent the group of persistent PCB congeners in those analyses and did not observe statistically significant correlations with any biomarkers of effects. DLC concentrations as measured by the DR-CALUX were also not correlated with biomarkers of effects. Age, gender, and alcohol consumption were not associated with the biomarkers of effects (data not shown).

**Figure 3:**
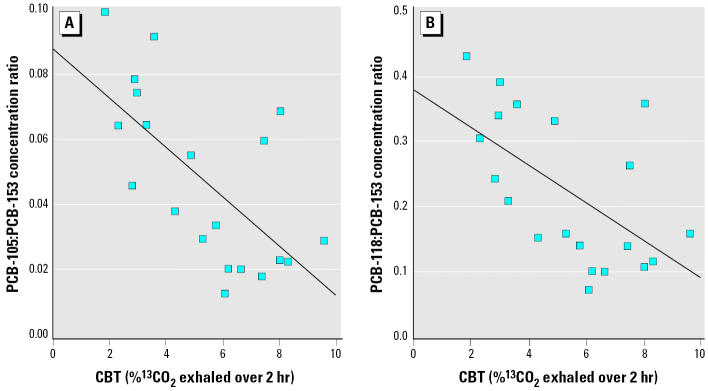
Inverse correlations between liver CYP1A2 activity (CBT values) and plasma concentrations of (A) PCB-105 (r = −0.62; p = 0.003) and (B) PCB-118 (r = −0.53; p = 0.02) in 20 residents of the Lower North Shore of the St. Lawrence River.

Next, we tested correlations between effects biomarkers and concentrations of mono-ortho PCB congeners 105 and 118, which did not exhibit strong collinearity with PCB-153. We found moderate inverse correlations between these mono-ortho congeners and the results from the CBT, which reached statistical significance for PCB-105 (p = 0.05). We hypothesized that the latter results may indicate increased biotransformation of PCB-105 and PCB-118 in participants with increased liver CYP1A2 activity. We further tested correlations between CYP1A2 activity and PCB-105: PCB-153 and PCB-118:PCB-153 concentration ratios to take into account the difference in PCB exposure between participants, which is best represented by concentrations of PCB-153 (a very persistent congener). We observed stronger negative correlations between CYP1A2 activity and these congener ratios than those noted above (Figure 3), further suggesting that PCB-105 and PCB-118 are biotransformed at an increased rate in participants exhibiting high CYP1A2 activities. Similar correlation coefficients were observed after stratifying for age (≤45, > 45 years) and gender (data not shown).

### Biomarkers of effects and smoking status.

Results for biomarkers of effects were stratified according to the smoking status because of the known effect of smoking on liver enzyme activities (Table 4). There was a tendency toward higher CBT values in smokers than in non-smokers, with the median value of smokers being 70% higher than that of nonsmokers (p = 0.07). The other biomarkers were not influenced by the smoking status of the participants.

## Discussion

We measured biomarkers of effects, more specifically, markers of hepatic microsomal enzyme induction, in residents of a remote coastal region of Québec who rely partly on sea products for sustenance. Our hypothesis was that biomarkers of hepatic enzyme induction would be increased as a result of the chronic exposure to OCs in this fish-eating population. Participants displayed high concentrations of several persistent, lipophilic compounds that are biomagnified in aquatic food chains such as PCBs, chlorinated pesticides, and DLCs. However, despite this high exposure, we did not find any statistically significant relations between biomarkers of OC exposure and markers of hepatic enzyme induction in this population.

Urinary d-glucaric has been suggested as a biomarker of effect induced by PCB exposure. Apostoli et al. (2003) reported a median concentration of 5.5 mmol/mol creatinine, with values ranging from 1.7 to 12.4 mmol/mol creatinine in 73 subjects who were exposed to PCBs through eating farm produces contaminated by runoff water from a nearby dielectric fluid plant. Somewhat lower values were obtained in our group: the median was 3.4 mmol/mol creatinine and the range spread from < 0.8 to 9.8 mmol/mol creatinine, when we excluded the two highest values displayed by subjects taking enzyme-inducing anticonvulsants. In results similar to ours, Apostoli et al. (2003) did not observe any relation between plasma PCB levels and urinary d-glucaric acid concentrations; smoking was also not related to this biomarker of enzyme induction. Median total PCB plasma concentration in their participants was 27 μg/L (range, 1.1–394 μg/L), compared with 18 μg/L (range, 2.5–61 μg/L) in our group. In workers with higher PCB exposure (total PCB plasma concentrations ranging from 88 to 1,359 μg/L), Maroni et al. (1984) noted a higher d-glucaric concentration than in an unexposed comparison group. However, the correlation of PCB plasma concentrations with d-glucaric concentrations was not statistically significant. Children and adults from Seveso, Italy, who were highly exposed to TCDD after its release to the atmosphere because of a malfunction in a chemical plant showed a statistically significant increase of d-glucaric acid elimination compared to the control groups, even 3 years after the accident (Ideo et al. 1985). Hence, although d-glucaric acid concentrations are elevated in urine samples of individuals who have been highly exposed to dioxins, it does not appear to be sensitive enough in situations involving low-level environmental PCB exposure.

With regard to urinary porphyrins, coproporphyrins were detected in all participants, whereas the other porphyrins were rarely if ever present in concentrations exceeding the detection limit of the analytical method (25 nmol/L). Furthermore, we did not observe any correlation between OCs and urinary coproporphyrin levels. Provided a sufficient exposure dose, several halogenated aromatic hydrocarbons can lead to increased urinary porphyrin excretion (Hogue and Brewster 1991). However, although acute poisoning and high occupational exposure to these compounds are clearly porphyrogenic (Daniell et al. 1997), environmental exposure has not been linked to porphyrinuria. A recent study conducted among 241 residents of Flix (Catalonia, Spain), a village located near an electrochemical factory where high atmospheric levels of HCB were detected, revealed lower urinary levels of coproporphyrins in participants exhibiting the highest HCB plasma levels (Sunyer et al. 2002). With regard to DLCs, Calvert et al. (1994) did not find an increased risk of showing out-of-range urinary uroporphyrin or coproporphyrin concentrations in U.S. workers exposed to TCDD, compared to referents. Moreover, a subgroup of 10 workers who had a mean serum TCDD concentration of 559 pg/g lipids (range, 769–1,593 pg/g lipids) at the time of the study had similar coproporphyrin levels as those of the referents (Calvert et al. 1994). Considering the lower exposure to DLCs documented in our fish-eating population (range, 37–287 pg TEQs/g lipids), the observed lack of porphyrogenic effect in relation to DLC exposure is consistent with evidence in the literature.

During the last 15 years, the CBT has been used to assess in vivo liver CYP1A2 activity in various population subgroups. Hepatic microsomal caffeine N-3-demethylation, the initial major step in caffeine biotransformation in humans, is selectively catalyzed by CYP1A2 (Butler et al. 1989). Among subjects without any known exposure to pharmaceutical or environmental enzyme inducers, CYP1A2 activity in smokers is 1.5–2 times that of non-smokers (Abraham et al. 2002; Kotake et al. 1982; Lambert et al. 1986). A group of 51 Michigan farmers, who were exposed to polybrominated biphenyls when a flame retardant was accidentally added to dairy cattle feed, showed a greater median CYP1A2 activity that that of unexposed Illinois residents (Lambert et al. 1990). The authors also reported a weak positive correlation between serum polybrominated biphenyl concentrations and CBT values, although close inspection of the data indicates that this correlation is mainly due to five individuals with high plasma concentrations (> 300 μg/L). The median CBT value for Taiwanese who were accidentally exposed to PCB/PCDF through the ingestion of contaminated rice oil (Yu-Cheng patients) was 440% higher than that of the unexposed control group (Lambert et al. 1992). In contrast, subjects exposed to TCDD after the Seveso incident did not exhibit high CBT values when tested 16–18 years after their exposure (Brambilla et al. 1995). Initially, concentrations of TCDD up to 6,300 ppt had been measured in plasma samples from these individuals, but contemporary levels were not mentioned by the authors. Abraham et al. (2002) also used the CBT to measure CYP1A2 activity in two women highly exposed to TCDD, one man moderately exposed, and 50 control subjects (30 nonsmokers and 20 smokers). Results indicated that in the women with very high TCDD exposure (> 10,000 ppt), CYP1A2 activity (as measured by the CBT) was at least 5 times the mean activity observed in non-smoking controls. The individual moderately exposed (1,000 ppt) exhibited a CYP1A2 activity similar to that of smokers in the control group, and at the high end of values displayed by nonsmoking controls (Abraham et al. 2002). With total DLC concentrations not exceeding 300 ppt in our participants, we conclude that their exposure level was too low to induce liver CYP1A2 activity above baseline, as measured by the CBT.

We observed a moderate correlation between liver CYP1A2 activities (CBT values) and urinary coproporphyrin concentrations in our participants. In the study by Sunyer et al. (2002) discussed above, smokers had a higher concentration of coproporphyrin III, which was the major coproporphyrin in urine samples. The authors mentioned that this finding agreed with a possible indirect effect of cigarette smoking through CYP1A2 induction. CYP1A2 has been involved in disturbances of porphyrin metabolism in mice but not in humans (Gorman et al. 2002; Sinclair et al. 2000). Our findings do support a possible link between altered porphyrin metabolism and liver CYP1A2 activity in humans.

A major strength of our study is the use of a cell-based assay, the DR-CALUX assay, to measure the total concentration of DLCs in plasma samples of participants. A moderate correlation was observed between results from this bioassay and the analytical chemistry method, indicating that the DR-CALUX assay is indeed responding to AhR agonists extracted from plasma samples. We did not expect a strong correlation with the analytical chemistry data because the bioassay integrates the contributions from all AhR agonists extracted from plasma, including several that are not measured by the HRGC/HRMS method that is, xenobiotics such as polycyclic aromatic hydrocarbons (Seidel et al. 2002), dietary components such as flavonoids (Amakura et al. 2003), or endogenous compounds such as bilirubin and biliverdin (Phelan et al. 1998). In addition, mixtures of DLCs extracted from the plasma may not induce responses that are additive in the DR-CALUX, unlike the assumption made by summing the contributions of all DLCs measured by analytical chemistry to yield a total TEQ concentration (Safe 1990).

Because of the small number of participants in this study, one could question its power to detect associations between effect biomarkers and exposure variables. With 20 subjects, power was sufficient to detect the fairly strong positive association between smoking and liver CYP1A2 activity (urinary cotinine concentrations vs. CBT values, r = 0.60; p = 0.005). In contrast, correlation coefficients between biomarkers of hepatic enzyme induction and biomarkers of exposure (DLCs and PCB-153) ranged from 0.07 to 0.23, indicating weak associations between these variables at best. Although we cannot rule out an effect of OC exposure on hepatic enzyme activity, our results suggest that, in this population, OC exposure is less important than smoking as an inducing factor of CYP1A2 activity. Similar results were obtained by Pereg et al. (2002), who measured CYP1A1 activity in placenta samples obtained from Inuit women giving birth in Nunavik (northern Québec, Canada). These authors noted a statistically significant relationship between placental CYP1A1 activity and smoking but not with OC body burden, hence suggesting, as in the present study, that environmental OC exposure was not high enough in this population to induce AhR-mediated effects.

The inverse correlation noted in the present study between liver CYP1A2 activity and plasma concentrations of PCB-105 and PCB-118 is of interest for two reasons. First, it supports a role for CYP1A2 in mediating the biotransformation of these mono-ortho congeners in humans. Brown et al. (1989) previously reported a particular profile of PCB congeners in plasma samples of PCDF-exposed individuals that featured relatively low concentrations of these mono-ortho congeners compared with concentrations of di-ortho congeners. Second, the negative relation of CYP1A2 to mono-ortho PCBs emphasizes the importance of considering other factors linked to CYP1A enzyme activities as possible causal agents whenever an association between plasma concentrations of these mono-ortho congeners and disease is observed in epidemiologic studies. Indeed, we expect plasma concentrations of these congeners in an individual to be influenced by dietary, environmental, or lifestyle exposures to compounds that either induce (i.e., polycyclic aromatic hydrocarbons in cigarette smoke) or inhibit CYP1A enzymes, or by functional genetic polymorphisms for these enzymes. Hence, the plasma concentration of mono-ortho PCBs might be a surrogate of CYP1A enzyme activity in the individual, the latter being directly or indirectly linked to the disease. In a recent case–control study in which we noted an association between breast cancer risk and plasma concentrations of mono-ortho PCBs (Demers et al. 2002), we speculated that the association could be explained by CYP1A enzyme activities in women altering both plasma concentrations of mono-*ortho* PCBs and estradiol levels, the latter being the causal agent.

In summary, we found no relation between biomarkers of OC exposure and markers of hepatic enzyme induction in this highly exposed group of fish eaters from the Lower North Shore of the St. Lawrence River. Our results suggest that smoking induces liver CYP1A2 activity, which in turn alters porphyrin metabolism and increases the biotransformation of mono-*ortho* PCBs.

## Figures and Tables

**Table 1:** Plasma OC concentrations in 40 residents of fish-eating communities along the Lower North Shore of the St. Lawrence River (Québec, Canada).

OC	Median	Arithmetic mean	SD	Minimum	Maximum
PCBs					
PCB-99	127	137	80	12	308
PCB-105	32	37	25	< 7^a^	107
PCB-118	145	167	103	32	437
PCB-138	533	553	274	36	1,164
PCB-153	766	817	398	90	1,668
PCB-156	87	91	51	15	218
PCB-170	151	167	91	24	359
PCB-180	629	645	361	85	1,447
PCB-183	64	70	35	< 7^a^	136
PCB-187	177	194	94	17	378
Total PCBs^c^	2,820	2,897	1,372	334	5,880
Chlorinated pesticides					
p,p′-DDE	1,086	1,681	1,576	56	6,262
HCB	104	101	42	35	223
Oxychlordane	43	53	48	< 7^b^	182
Mirex	46	47	32	< 7^b^	115
trans-Nonachlor	87	98	47	16	223
DLCs^c^	93	102	57	37	287

PCBs and chlorinated pesticides were analyzed by HRGC/ECD and are reported in units of microgram per kilogram lipids.

a The limit of detection is 0.5 μg/L, which corresponds to approximately 7 μg/kg lipids.

b Sum of 13 congeners.

c DLCs were determined by the DR-CALUX bioassay and are reported as nanogram TEQs per kilogram lipids.

**Table 2:** Biomarkers of effects in 40 residents of fish-eating communities along the Lower North Shore of the St. Lawrence River (Québec, Canada).

Biomarkers	No. detected	Median	Arithmetic mean	SD	Minimum	Maximum
d-Glucaric acid (mmol/mol creatinine)	38	3.4	4.4	4.5	< 0.8	26.0
Uroporphyrins (μmol/mol creatinine)	6	1.2	1.2	0.4	< 0.4	2.2
Coproporphyrins (μmol/mol creatinine)	40	11.0	11.5	3.5	5.7	19.2
CBT (% total)^a^	21^b^	5.3	5.4	2.2	1.9	9.6

a Percentage of total 13C dose exhaled as 13CO_2_ over a 2-hr period.

b The CBT was administered to the 40 participants, but for 19 of them CO_2_ concentrations in air samples were too low for isotopic analysis to be performed.

**Table 3:** Correlations^a^ between biomarkers of exposure and biomarkers of effects in residents of fish-eating communities along the Lower North Shore of the St. Lawrence River (Québec, Canada).

	Urinary d-glucaric acid (n = 38)		Urinary coproporphyrins (n = 38)		CBT (n = 20)	
Biomarker exposure/effects	r	p-Value	r	p-Value	r	p-Value
Cotinine	0.28	0.09	0.10	0.57	0.60	0.005
PCB-153	0.20	0.22	0.06	0.73	0.07	0.76
PCB-105	−0.05	0.75	−0.24	0.15	−0.45	0.05
PCB-118	0.01	0.99	−0.22	0.18	−0.41	0.07
DLCs	0.23	0.18	0.01	0.98	0.12	0.61

a Spearman’s correlation coefficient. Correlation analyses were performed after excluding two participants taking anticonvulsants.

**Table 4:** Biomarkers of effects in relation to the smoking status in residents of fish-eating communities along the Lower North Shore of the St. Lawrence River (Québec, Canada).

	Nonsmokers				Smokers				
Biomarker	Median	Mean	SD	n	Median	Mean	SD	n	p-Value^a^
d-Glucaric acid (mmol/mol creatinine)	3.6	3.6	1.7	21	2.9	3.6	2.6	17	0.33
Coproporphyrins (μmol/mol creatinine)	9.8	10.7	3.3	21	11.3	11.7	2.9	17	0.28
CBT^b^	3.6	4.7	2.3	13	6.1	6.7	1.8	7	0.07

a p-Value for Mann-Whitney U-test. Comparisons were effected after excluding two individuals taking anticonvulsants.

b Results of the CBT are expressed as the percent of total 13C dose exhaled as 13CO_2_ over a 2-hr period.
